# Distributed Optical Fiber Sensors for Monitoring of Civil Engineering Structures

**DOI:** 10.3390/s22124368

**Published:** 2022-06-09

**Authors:** Kinzo Kishida, Michio Imai, Junichi Kawabata, Artur Guzik

**Affiliations:** 1 Neubrex Co., Ltd., 1-1-24 Sakaemachi-dori, Kobe 650-0024, Japan; 2 Kajima Co., 1-3-8 Motoakasaka, Minatoku, Tokyo 107-8477, Japan; michio@kajima.com (M.I.); kawabata-j@kajima.com (J.K.); 3 Neubrex Infra AG, Badstrasse 4, 5400 Baden, Switzerland; guzik@neubrex.com

**Keywords:** civil engineering, DFOS, Rayleigh frequency shift, distributed RIP

## Abstract

Distributed Fiber Optics Sensing (DFOS) is a mature technology, with known, tested, verified, and even certified performance of various interrogators and measurement methods, which include Distributed Temperature Sensing (DTS), Distributed Temperature-Strain Sensing (DTSS), and Distributed Acoustic Sensing (DAS). This paper reviews recent progress in two critical areas of DFOS implementation in large scale civil engineering structures. First is the substantial improvement in sensing accuracy achieved by replacing Brillouin scattering-based measurements with its Rayleigh counterpart. The second is progress in acquisition speed and robustness, as now engineers can observe parameters of interest in real-time, and make informed, operational decisions regarding quality and safety. This received a high valuation from field engineers when used during the construction stage of the project. Furthermore, this change in the use of DFOS in civil engineering greatly increases the practical possibility of installing FO cables permanently. The same FO cables can be later used for long-term monitoring, during maintenance periods throughout the structure’s lifetime. To illustrate these two advances, we present a comparison between Brillouin and Rayleigh scattering measurements, and their accuracy, and highlight the importance of temperature and strain separation. We also present several important applications in large scale civil engineering infrastructure projects.

## 1. Introduction

From the very early stages of Distributed Fiber Optics Sensing (DFOS) development and deployment, the Structural Health Monitoring (SHM) of critical infrastructure has been considered as a valuable use of the technology. It promised to replace point or point-wise sensors and continuously monitor values of strain and/or temperature. This long-term monitoring, during the maintenance period of a structure’s lifetime, remained for decades the almost exclusive use of the technology. However, this meant that the intended users of the technology were different than those who were deploying and installing the systems, as the best, and sometimes the only time to permanently install sensing fibers was during the construction stage. Due to long acquisition times, limited spatial resolution, and sensitivity not meeting certain technical requirements, there was limited interest from the construction industry in using distributed fiber optic sensing (DFOS) systems for construction engineering data acquisition and analysis. The situation in civil engineering changed with the substantial improvement in DFOS technology, in sensing cable and interrogators performance, which reshaped the landscape of DFOS-based Structural Health Monitoring.

**Figure 1:**
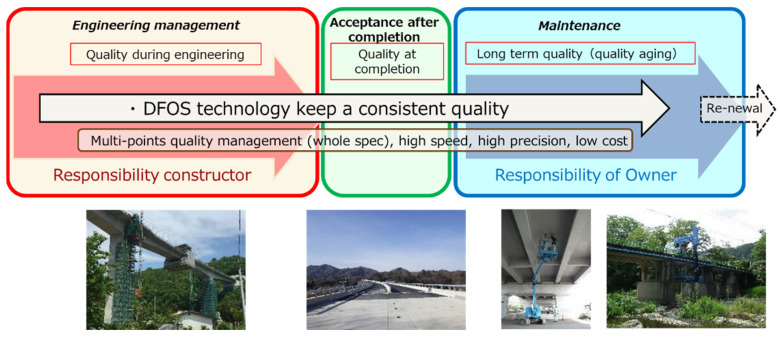
DFOS is a technology for the entire life cycle of a structure’s monitoring.

With current technology, one can utilize DFOS during the engineering stage, allowing its use for the entire life cycle of the structure. This is schematically presented in Figure 1.

In 2021, a DFOS manual for civil engineering was published by Photonic Sensing Consortium in Japan (PhoSC, https://phosc.jp/ [1]). PhoSc brings together construction companies and fiber optical sensing specialists and companies. PhoSC maintains one of the best technology introduction sites, where basic principle and field technologies are explained [1].

**Figure 2:**
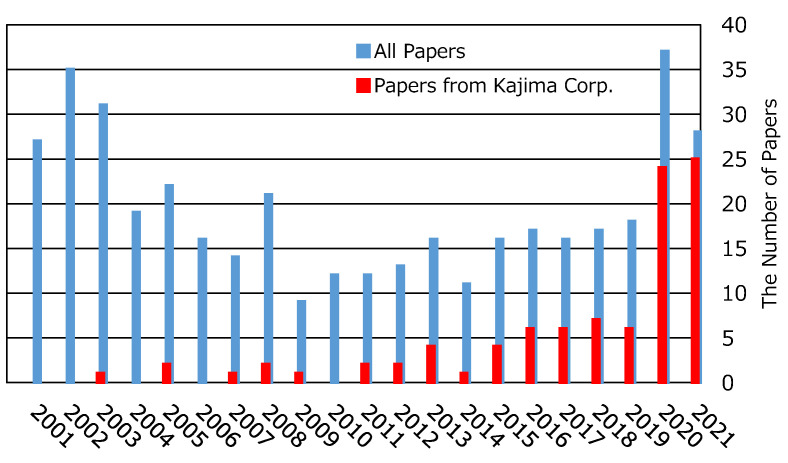
Change in DFOS publications in the Japanese Civil Engineering Association.

The growing interest in using DFOS is also exhibited in the number of papers related to DFOS in the Japanese Civil Engineering Association. It has rapidly increased in the last 3 years, as shown in Figure 2, where red bars denote the publications from Kajima Co. (Tokyo, Japan). There is already a number of major projects, in which DFOS technology will be deployed to monitor critical parameters during their engineering phase. The total estimated budget for these projects is USD 2000 MM or more over the next 3 to 10 years.

For the use of interrogators in the engineering phase of the construction, based on gathered experience from a wide range of users and on a variety of projects types, the following a DFOS system characteristics are required for a successful project:

- Strain precision should be less than 1 µε;
- Precision should not degrade with distance;
- Spatial resolution should be better than 20 cm,

The list of secondary DFOS system characteristics includes:

- Acquisition time, for a distance up to 500 m, should be shorter than 5 s;
- Allow for mechanical strain-thermal strain and temperature separation;
- Be reliable and utilize low-cost FO sensing cables.

These system characteristics are essentially already met in oil and gas industry applications [2,3]. The authors in [4] have a good description of the technology.

This work presents key technologies used in civil engineering, namely, Rayleigh and Brillouin backscattering-based strain and temperature sensing. This work does not provide a full list of the interrogator research papers, and only concentrates on two types, which meet the critical as well as some of the less-essential characteristic requirements for the interrogator types listed above.

The references are selected mainly for understanding by field engineers, without a background in photonics. Interested readers can find many DFOS review papers, such as [5], which contains over 150 references.

The technology called Distributed Rayleigh Intensity Pattern (RIP) technology [4] is discussed first. In earlier publications, this method was referred to as Tunable-Wavelength Coherent Optical Time Domain Reflectometry (TW-CODTR) [6,7]. The second type of technology is referred to as Pulse-Shift-Pulse Brillouin Optical Time Domain Reflectometry (PSP-BOTDR). The publication in English is in preparation, while the original is presented in [8]. The references include a detailed description of sensing principles, interrogator performance parameters, and their schematic diagrams.

While this paper is intended to be a guideline for field engineers, and not a review of interrogation methods and instruments, we still summarized in Table 1 the values of essential performance parameters for the most frequently used and commercially available DFOS measurement methods. The values listed in Table 1 should be compared with the civil engineering project requirements listed and discussed above.

In Table 1, BOTDR stands for Brillouin Optical Time Domain Reflectometry, which requires single-end fiber access only, while BOTDA for Brillouin Optical Time Domain Analysis, which is a double-end access technology. The double-end access requirement alone limits the practical use of the technique, making fiber installation far more challenging, and most importantly no signal can be acquired in case of any fiber break. There are several related measurement techniques, including Pulse-Pre-Pump (PPP-BOTDA), which we used in one of the verification tests reported in this paper. Those and other sensing techniques have already been reviewed in a number of papers, including [5].

In the authors’ experience when using OBR (OFDR)-based measurements methods, if the frequency scan range is large, even in a laboratory setup for strain sensing, the accuracy can drop to 5 µε. Authors are also unaware of publications examining the accuracy or describing the experimental setup and procedures to verify these results.

## 2. Principle and Performance of Distributed RIP Method and Comparison with Brillouin Scattering

The first implementation of Rayleigh-type backscattering for strain measurements was developed in the form of Optical Frequency Domain Reflectometry (OFDR) and presented in [9]. While this method provides data with an unprecedented millimeters-order spatial resolution, the relatively short distance range of below 100 m limits the usage of the method in large-scale SHM applications. Moreover, any changes to fiber overall length, such as adding a patch cord, make it impossible for the method to determine the strain change. This feature alone makes the use of this technique in long-term monitoring projects challenging at best.

An alternative approach, using acquisition in the time domain based on Coherent Optical Time Domain Reflectometry (C-OTDR) was proposed in [6]. The method offers lower, cm-order, and spatial resolution, but importantly provides measurements over very long distances. Implementation of this method using a tunable wavelength (TW-COTDR) laser and frequency scanning to obtain power spectrum was presented in [7]. The advantage of this technology has proven to be its very high precision, which does not change with spatial resolution. Recently, it was realized that the signal used in TW-COTDR is the Rayleigh Intensity Pattern (RIP), which is the basic optic fiber backscattering phenomenon observed in any type of fiber optics. RIP does not relate to the phase of the optic signal, but changes with spatial resolution.

It is emphasized here that both frequency and time-domain implementations of the Rayleigh-based scattering method provide relative results/changes in strain and/or temperature between an arbitrarily selected reference state and a given instance measurement. The measured frequency shift is obtained by cross-correlation. The frequency shift, ${\Delta \nu }_{R}$, induced by the strain/temperature changes can be expressed using:

(1) $${\Delta \nu }_{R}=C_{21}\;\Delta \varepsilon +C_{22}\;\Delta T$$

where Δε and ΔT are strain and temperature changes, respectively, while $C_{21}$ and $C_{22}$ correspond to the frequency-strain and frequency-temperature conversion coefficients.

**Figure 3:**
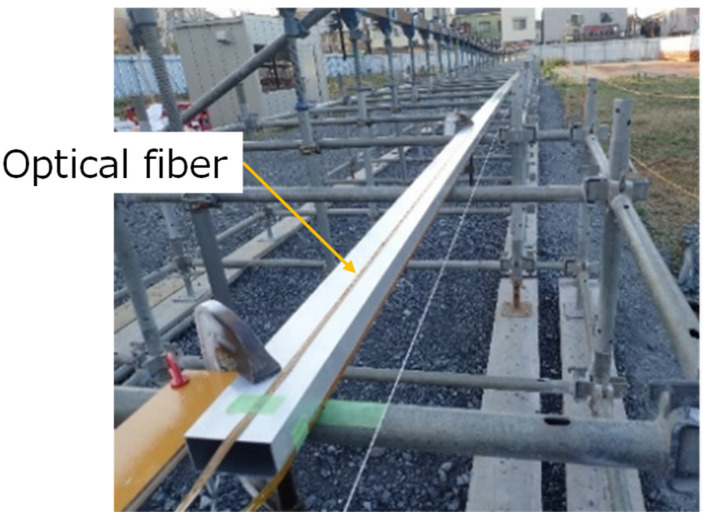
The fiber layout and test specimen.

**Figure 4:**
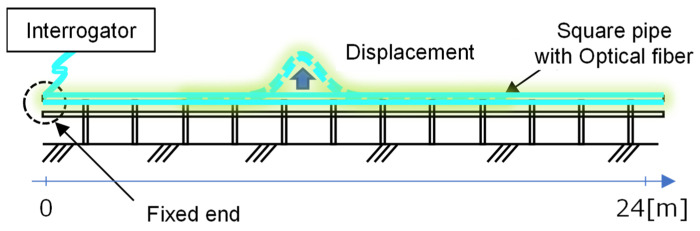
Fiber layout and loading test schematic.

The sensitivity of Rayleigh scattering is approximately three orders of magnitude higher than Brillouin-based sensing methods. For RIP measured using TW-COTDR, the achievable precision for strain is as high as 0.078 micro-strains or 0.01 K for the temperature. This leads to substantial improvements in the derived results, such as displacements. This is most evident when contrasting traditional Brillouin measurements with TW-COTDR results. The simple test with a 24 m long, multi-point supported beam and enforced displacements applied at a 10 m distance was arranged, as shown in Figure 3 and Figure 4.

**Figure 5:**
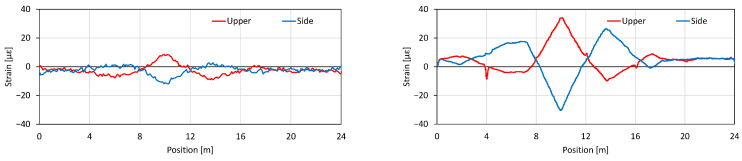
Measured strain distribution along the fiber: Brillouin (**left**) and Rayleigh (**right**).

The measured strain changes along the beam are plotted in Figure 5, where on the left is the distribution measured with the Brillouin technique, and on the right, the same with Rayleigh scattering.

Both results show different strains, positive and negative values, at the applied displacement point. The higher resolution and sensitivity of the new technique means that the strains measured there are more accurate, as the lower resolution always underestimates localized strain changes.

**Figure 6:**
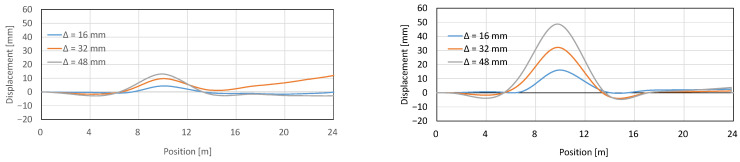
Obtained displacement (deflection) along the fiber: Brillouin (**left**) and Rayleigh (**right**) verified by displacement measurements.

Figure 6 presents the derived result of the displacement distribution that is obtained by integrating from the origin the measured strain distribution. The displacements obtained via Brillouin-based strain measurements are again substantially different than that actually applied to the beam, while the one calculated based on Rayleigh measurements is almost exact. This is even more visible at the very end of the beam, at 24 m, where, due to beam support, there is no beam displacement. The displacement based on Rayleigh data gives, again, the correct value there, while Brillouin values are as if a large deflection occurred, which is clearly incorrect. This significant difference between the two results is due to the fact that the conversion to displacements is cumulative, and the errors are propagated in the process. This is one of the applications, where the advantages of the new measurement technology can be used.

The high sensitivity and spatial resolution of the TW-COTDR technique are key to its success in various other applications, including well drilling [10], landslide monitoring [11], or water migration in rocks [12]. The only weaknesses of TW-COTDR are that it is relatively slow and that the non-uniform distribution of strain and/or temperature within the spatial resolution leads to low correlation values and, therefore, difficulties in determining the shift value. Additionally, in many applications, an absolute distribution of strain value is also of great importance. In such cases, only Brillouin-based techniques offer these types of results.

## 3. PSP-BOTDR for Measuring Temperature and Strain in Single Fiber

Brillouin-type interrogators cannot achieve precision comparable to distributed RIP. However, newly developed, and commercialized PSP-BOTDR is introduced here due to its outstanding performance as:

- It requires only a single end fiber access;
- Reaches the resolution of 10 cm with sensing distance up to 10 km;
- Its precision of the measurement does not change with distance and has no drift along all the measurement ranges.

In BOTDR, the Brillouin frequency shift (BFS) is determined from the center frequency of the Brillouin spectrum for a pulse. There is, however, an uncertain relationship between the peak width of the spectrum and the spatial resolution [8], which, in principle, limits the performance. There were already several methods proposed to overcome this limitation and obtain high (cm-order) spatial resolution with single-end fiber access. One of the methods developed is Pulse-Shift-Pulse BOTDR [13,14].

The PSP-BOTDR uses a cross-spectrum of short and long pulses, which do not overlap but have different light phases. The resulting cross-spectrum has a linewidth of 50 MHz, which is not much wider than an ideal, Lorentz spectrum of 30 MHz. This allows PSP-BOTDR to achieve a very high spatial resolution of 10 cm. The technology has already been commercialized with the release of the NBX-8021 interrogator unit (IU) [8]. The main measurement technique in the instrument is encoded PSP-BOTDR, but it is also equipped with its non-coded PSP-BOTDR and conventional BOTDR versions. All types of acquisitions are based on wide-band reception. Measurement time is shown in Table 1. For the coded PSP-BOTDR, the measurement time is 7 min for an average of 2^15^ times at a distance of 10 km and with a sampling interval of 10 cm.

**Figure 7:**
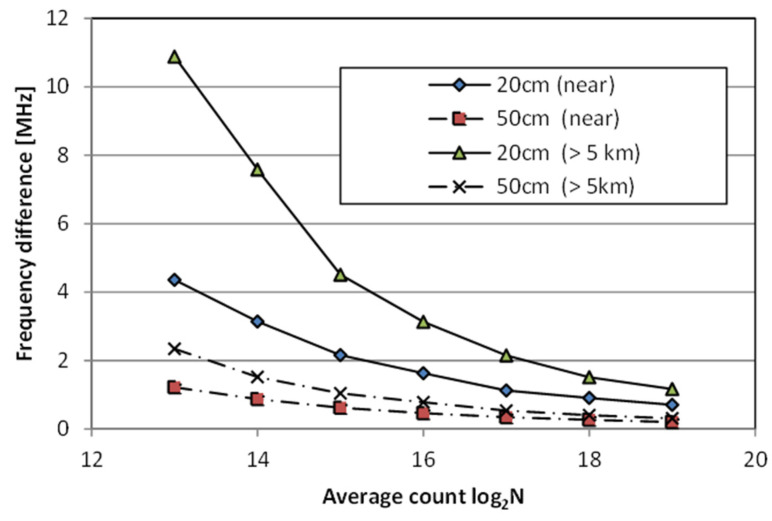
NBX-8100 spatial resolution performance. 1 MHz means 20 με.

With longer acquisition times, the standard deviation of measurement data decreases, reaching 6 µε even at 20 cm resolution, which is the highest level for Brillouin technology available today. As shown in Figure 7, the accuracy decreases according to the theoretically expected value ∝ 1/(average number of times)1/2.

**Figure 8:**
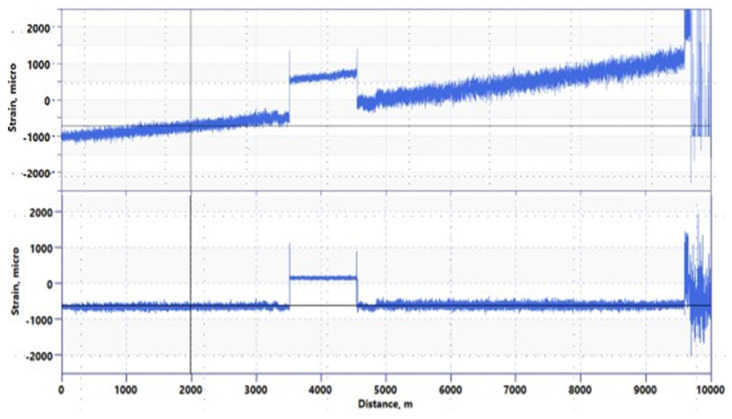
Comparison of Coded PSP and conventional BOTDR (using 2^18^ average count at 20 cm SR).

Figure 8 shows a comparison between the PSP-BOTDR and the conventional BOTDR measured over a 10 km distance. The PSP-BOTDR accurately measures the Brillouin center frequency with almost the same accuracy over the entire 10 km range. Strain distribution for conventional BOTDR indicates non-linear effects of the backscattering and, thus, a very inaccurate measurement.

PSP-BOTDR provides a reliable Brillouin center frequency shift:

(2) $${\Delta \nu }_{B}=C_{11}\;\Delta \varepsilon +C_{12}\;\Delta T$$

where Δε and ΔT are strain and temperature changes, respectively, as in Equation (1), while $C_{11}$ and $C_{12}$ correspond to the frequency-strain and frequency-temperature conversion coefficients. By solving the set of Equations (1) and (2), as proposed in 2013 by [7], temperature and strain can be separated, providing the strain and temperature values along the single fiber.

The accuracy of such an approach is clearly demonstrated when compared with traditional DTS measurements. As an example, we present the temperature obtained after 7 years of continuous monitoring of permanently embedded fibers. In this application, there are multi-mode (MM) and single-mode (SM) fibers in the same sensing cable, and additionally, there are water tanks on both sides of the fiber loop for DTS calibration. The Brillouin IU used is PPP-BOTDA [15,16]. The drift of center frequency along the distance does not pose a problem here, as the distance is short.

**Figure 9:**
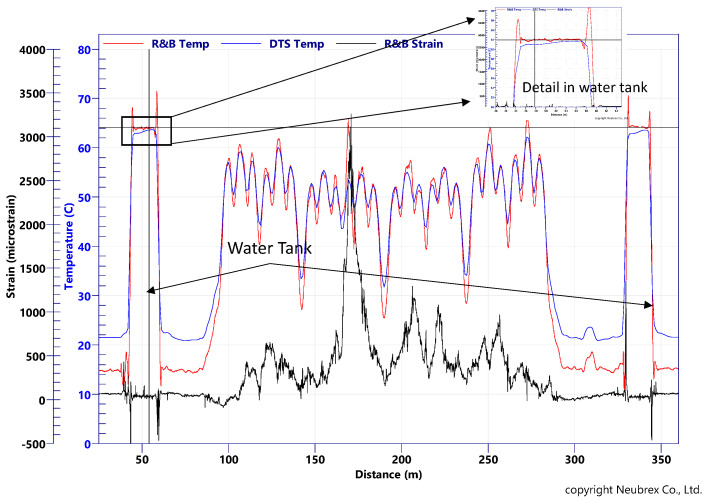
Strain and temperature separated in comparison with DTS data.

The hybrid Rayleigh–Brillouin method cannot reach accuracy better than 0.3 °C, as it is limited by the precision of Brillouin, as shown in Figure 9. It offers, however, a powerful tool for field applications, as the only requirement there is an SM fiber with single-end access. The required fiber-related coefficient, C_ij_, can be easily obtained experimentally via calibration.

## 4. Application Examples

In this section, we present selected application examples from various deployments of DFOS during the construction (engineering) phase of civil infrastructure. In each deployment, the TW-COTDR and PSP-BOTDR methods introduced earlier were used for data acquisition.

### 4.1. Monitoring of Caissons Deformations during Construction

**Figure 10:**
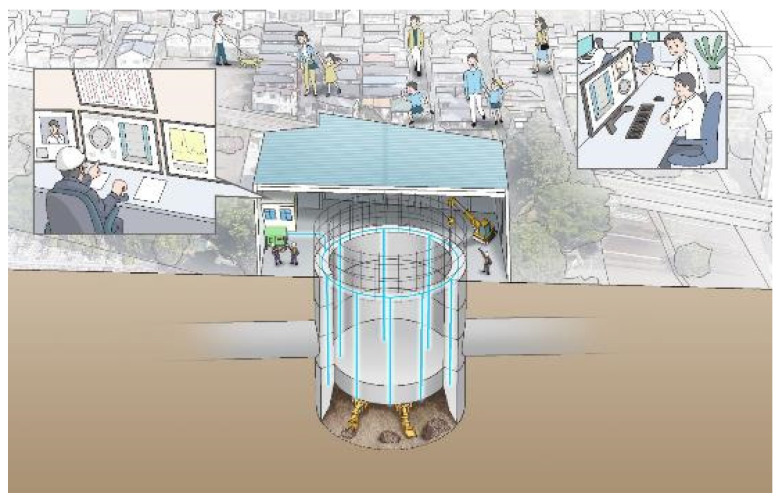
Example of open-type caisson during construction.

**Figure 11:**
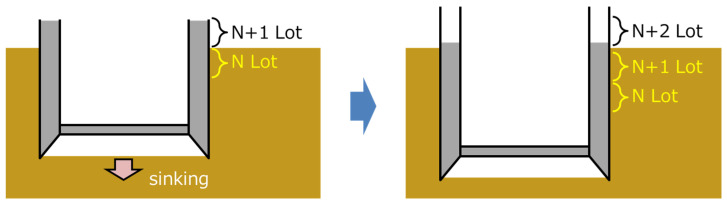
Stages of caisson construction.

A caisson is a box- or cylindrical- shaped, watertight structure, usually made of reinforced concrete and built first on the ground and then installed by sinking it into the ground while excavating the lower part. An example of such construction is shown in Figure 10. Figure 11 schematically presents the sequential and repeatable process always involved in the construction. For each lot, reinforcing steel and formwork are assembled, and concrete is poured, cured, de-framed, and sunk. This process is repeated for each lot.

Once excavation starts, it is important to balance the self-weight of the structure, the reaction forces between the caisson edge at the bottom and the ground, and the frictional forces between the ground and the structure. In essence, the concrete frame is subjected to compressive strains due to its own weight. However, if there is excessive friction due to clamping or eccentricity from the surrounding ground, the caisson cannot be sunk and is suspended or torqued. This can lead to cracking or sudden settlement impacts, which can have a detrimental effect on the surrounding area. In the event of tightening, a material called lubricant is applied to reduce the frictional forces between the ground and the structure. If the strain state can be visualized in real time by installing optical fibers in the structure, it is possible to determine the appropriate timing and location of the injection based on actual, real-time data, and reflect this in the next action.

**Figure 12:**
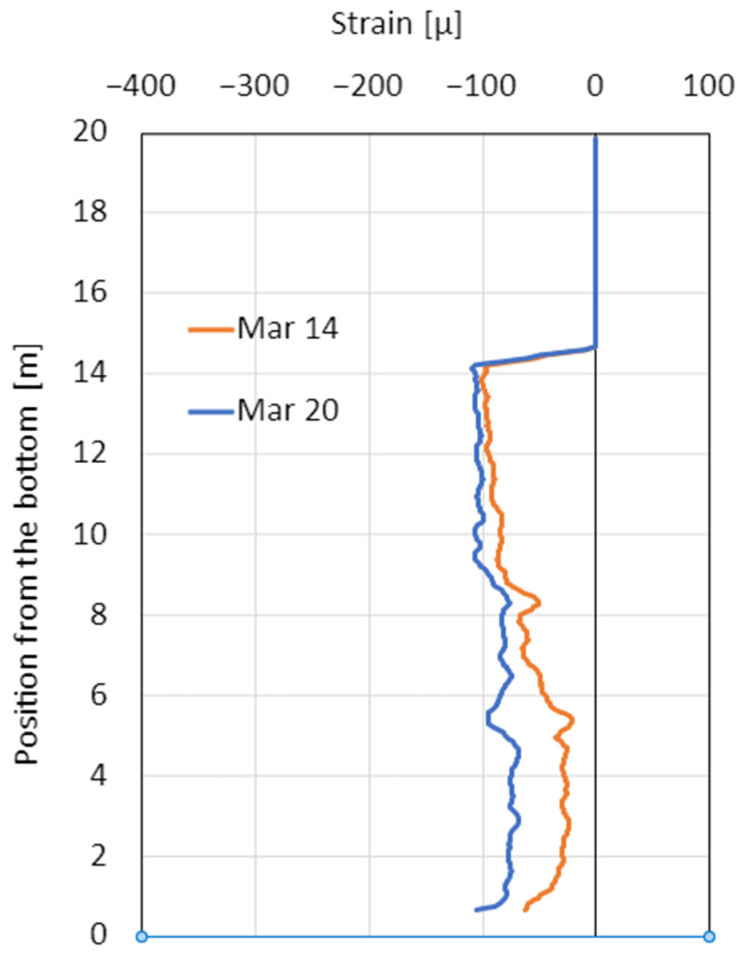
Strain traces at selected time along the caisson.

With the installed optical fiber, it is possible to pinpoint the location of abnormal strains. An example of such measurements is presented in Figure 12.

The vertical axis shows the position from the base and the horizontal axis shows the strain, with tension on the right and compression on the left. In the strain distribution obtained on 14 May, the orange line shows portions of the structure in tension, while for data on 20 May, the blue line indicates that the frictional force was reduced there, after the addition of the lubricant. These results are plotted in real time, so that the latest data can be shared and continuously checked, whether in the caisson operation room or in a remote-control room. For example, if the threshold value is set at +50 µε before cracks appear, an alarm can be displayed when the value is exceeded. This allows the sinking of caissons to be carried out with experience and intuition, but with objective data to determine where and when to add lubricant, resulting in high-quality construction with minimal impact on the surrounding environment and construction.

### 4.2. Underground Displacements Measurements

One of the applications that especially requires high-precision and high-resolution strain distribution measurement is deformation detection, which uses strain distribution data and converts them to displacement values. This is possible by attaching several optical fibers to a rod-like object. The displacement is calculated from the measured bending strain and integrating it from a fixed point/end.

The advantages of distributed measurements over the conventional discrete measurement of underground displacements, such as inclinometers, make it possible to realize a highly accurate and continuous underground displacement measuring device. It is also usually easy to construct, as the pipe is small and light, and the diameter of the borehole can be reduced during installation. Moreover, unlike in point measurements, the overall cost does not depend on the number of measuring points.

**Figure 13:**
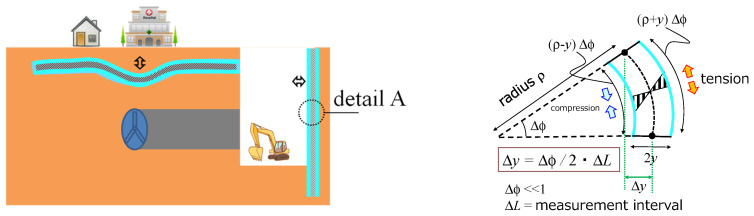
Displacement measurements (case without axial force).

Assuming that axial force is negligible, the deflection estimation can be performed in real-time and during the engineering phase of the construction. The mechanical assumptions are schematically presented in Figure 13, right. The principles of such deflection estimation can be applied and used to determine the deformation of the retaining wall.

**Figure 14:**
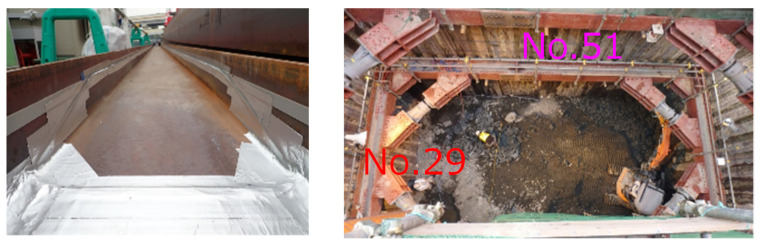
Displacement measurements, installed fiber (**left**), and construction site (**right**).

As shown in Figure 14 left, an optical fiber was attached to the flange of the earth retaining steel. As shown in the center, the displacements associated with excavation were measured with optical fiber using No. 29 and No. 51 earth-retaining steel supports and compared with conventional inclinometers.

**Figure 15:**
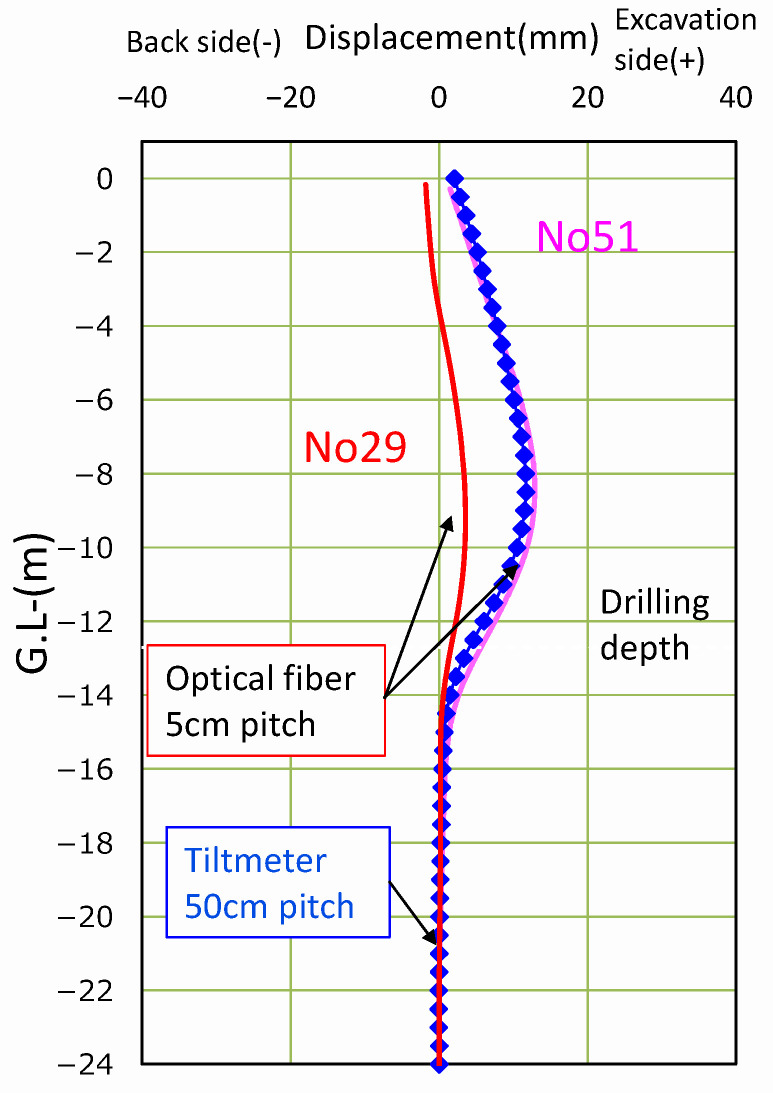
Displacement measurements: inclinometers and optical fibers data.

The results are shown in Figure 15. The vertical axis indicates the position in the depth direction, and the horizontal axis obtained displacement. The red line is the result of No. 29 and the pink line is the result of No. 51 optical fibers. The excavation causes a slight deformation towards the excavation side, centering between 8 and 10 m. The blue line is the displacement obtained by the inclinometer.

With optical fiber, we were able to grasp the results equivalent to those of inclinometers in more detail than ever before. Those valuable details can be used to determine whether the safety factor used in design and construction is required or is overestimated. With simple installation, it is possible to provide feedback on the temporary design and construction that achieves both economic efficiency and safety.

### 4.3. Tunnel Excavation Monitoring

**Figure 16:**
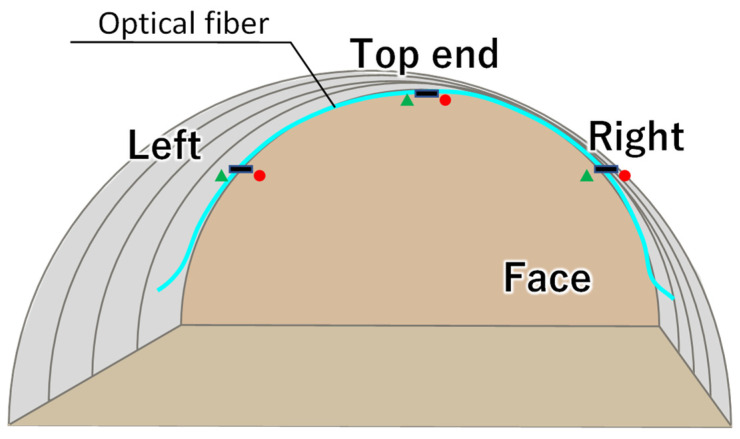
Optical fiber layout and location of gauges.

The application of the DFOS for monitoring tunnel excavation is one typical example of technology deployment. This time measurements were obtained in real-time and used during construction to ensure its safety. The fiber layout and location of strain gauges, used for validation purposes, are presented in Figure 16.

**Figure 17:**
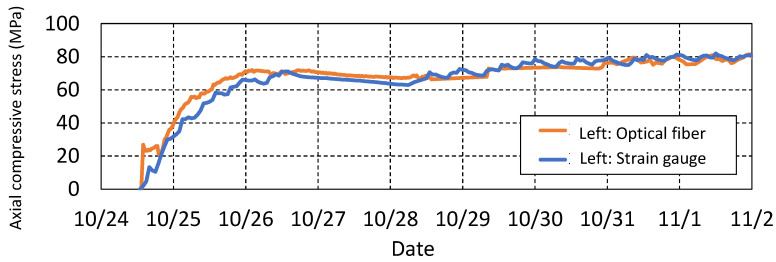
Comparison of optical fiber and strain gauges (at gauge location) as a function of time.

**Figure 18:**
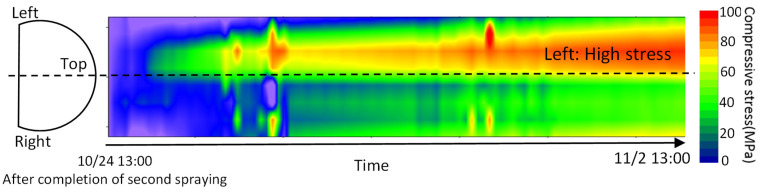
Optical fiber measurement results—waterfall plot of stress distribution.

The measurement results are shown in Figure 17. The stress, as a function of time at the location of the gauge on the left shoulder, is compared with the gauge data. It demonstrates that optical fiber matches well with the strain gauge. Figure 18 presents the change in the stress distribution over time measured by the optical fiber. The more intensive the red color, the greater the stress. Optical fiber enables such comprehensive visualization.

**Figure 19:**
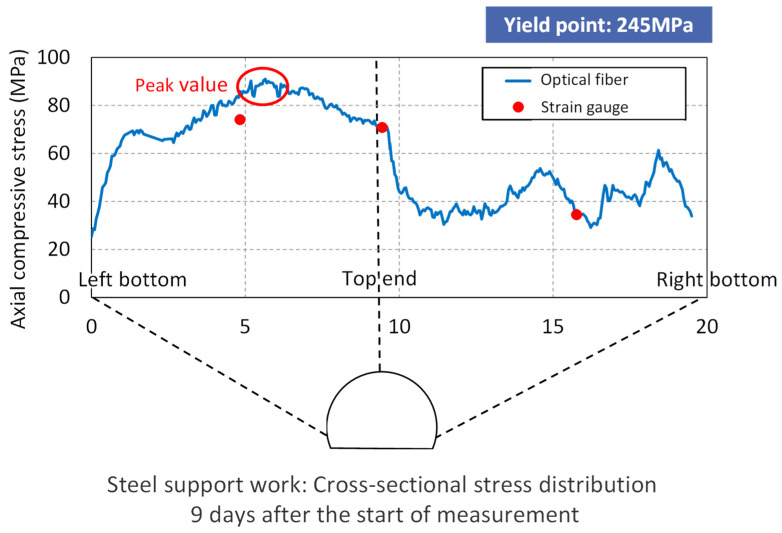
Optical fiber measurement results.

Finally, Figure 19 shows the cross-sectional stress distribution 9 days after the start of measurement. Blue is the optical fiber data and red dots are the values from strain gauges.

With optical fiber, high-resolution data can be used to understand the overall stress distribution and the ground pressure distribution, and the peak stress value is not missed. It is possible to grasp the time change after excavation, select an appropriate support pattern, realize efficient construction by selecting a support that does not become excessive, and realize safe construction that prevents collapse. Naturally, with the installed fiber remaining fixed in the tunnel, it provides means for maintenance-type measurements during the service period.

### 4.4. Construction Management: Concrete Frame

The final case is concrete filling detection. When constructing concrete, vibration is applied with a vibrator to improve filling so that the concrete will spread evenly after being poured in. However, it was difficult to visually confirm the filled concrete that corresponds to the excavated part of the caisson and the lining concrete of the tunnel as shown in the illustration in the middle, and there was a problem in filling it.

As a conventional technology, the filling detection sensor and RI tester (measurement of water content by neutron beam) can confirm the filling only at selected points, and the method of using a transparent formwork is limited to a visible/reachable place. These are the main challenges during construction. In addition, it is not possible to detect during construction by driving concrete with an electromagnetic wave radar.

**Figure 20:**
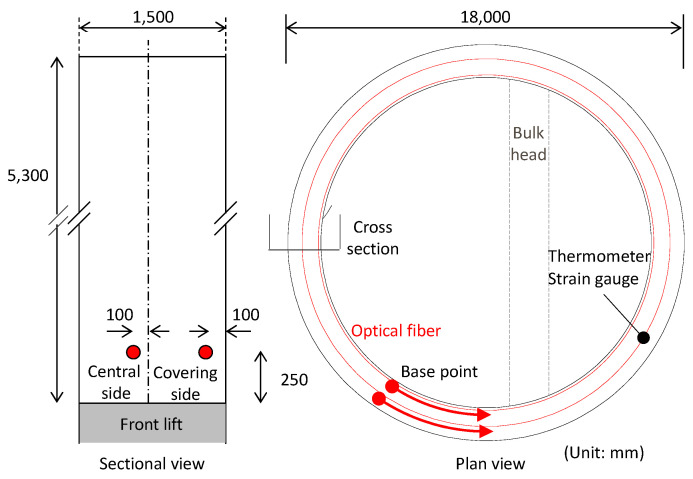
Concrete frame dimensions.

**Figure 21:**
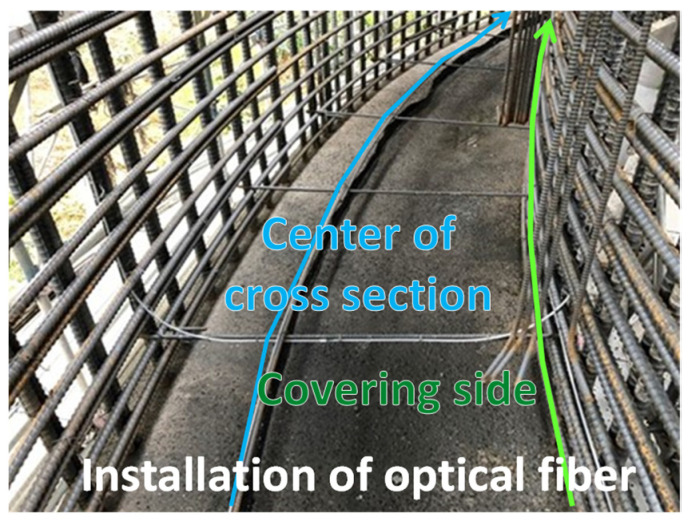
Concrete frame during construction and location of optical fibers.

Therefore, we tried to detect concrete filling using a distributed optical fiber sensor [17]. The dimensions of the frame are presented in Figure 20, while the frame during construction is shown in Figure 21.

**Figure 22:**
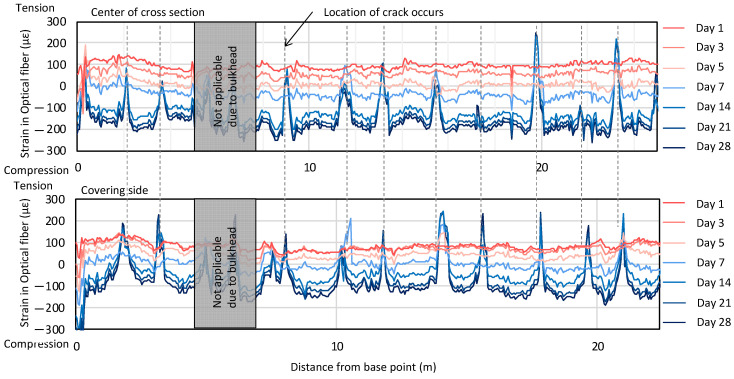
Strain change distribution as a function of time.

The measurement results are presented in Figure 22. The upper plot shows the change in strain over time. The results obtained with high spatial resolution enable one to detect the location of the tracks, which form over time, when the concrete cures. The location of the cracks is indicated by vertical dashed lines.

### 4.5. Correction of DTS Using PSP-BOTDR

One of the features of the Brillouin measurement is the ability to obtain an absolute value of strain and/or temperature. Even though it is the only way to measure absolute values, the frequency drift with the distance of the conventional BOTDR limits its application in the field. Figure 9 shows an example of the PSP-BOTDR, in which this drift is eliminated. The results of Distributed Temperature Sensing (DTS) and PSP-BOTDR measured in an oil well are shown.

The DTS temperature measurement requires calibration using a parameter that depends on the optical fiber (the ratio of optical attenuation between distant wavelengths). However, it is difficult to obtain the exact value of this parameter in advance.

**Figure 23:**
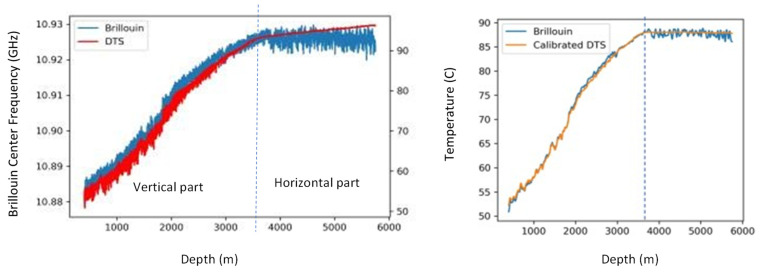
Application of PSP-BOTDR to calibrate DTS measurements.

In Figure 23, the DTS temperature measurement results on the left show that there is a slope in the horizontal well, where the temperature should be the same, and that the calibration error of the DTS increases with distance. The measured value of the DTS in the same well is the Brillouin frequency shift due to both strain and temperature, but since the strain is a local variation, the temperature change can be obtained by smoothing. When the DTS calibration was corrected using the Brillouin-measured temperature, a good agreement was observed over a long interval as shown in Figure 23 on the right. On-site temperature calibration of DTS has been a challenge for many years but has now been made possible by using the results of the PSP-BOTDR measurements.

### 4.6. Cement Injection Monitoring

Finally, we present an application of optical fiber sensing to monitor the quality of concrete injection. Ensuring that injected concrete is uniformly distributed, and no voids created, is a very common engineering problem, from construction and civil engineering to the oil and gas industry. The difficulty arises from the fact that the filling status after pouring/injecting concrete cannot be visually confirmed, and is instead determined by the filling detection sensor or RI tester. Most of the methods used, however, check only the local filling status, at the point of installation. Far more reliable verification can be achieved using electromagnetic wave radar, but it can be performed only after the curing process. In general, all of the methods currently used for verification of concrete injection quality are post-factum and/or very difficult to use in real-time during injection.

Optical fiber sensing offers a huge advantage over traditional methods, as it can provide distributed data, covering a larger space of the structure, and provide them in real-time. DFOS was used before for void detection but usually in the form of temperature (DTS) measurements. This approach, however, is also limited to measuring temperature changes due to curing. In many cases the results of voids detection were inconclusive. Small voids produced only small, local temperature changes, which were smoothed out, due to the low spatial resolution of DTS interrogators.

**Figure 24:**
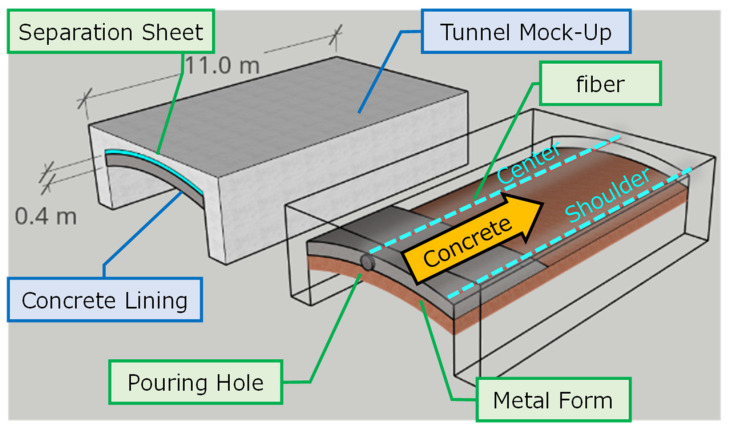
Mockup tunnel dimensions and locations of installed sensing fibers.

We proposed to use DFOS and strain change measurements to detect, in real-time, the location of the voids. Kajima Co. decided to test this procedure not only in the lab, but in large-scale, controlled experiments. For this purpose, the 11 m long tunnel mockup was constructed, with 40 cm concrete lining. Two sensing fibers were installed in the center and shoulder of the frame. The mockup dimensions and locations of the fibers are presented in Figure 24.

**Figure 25:**
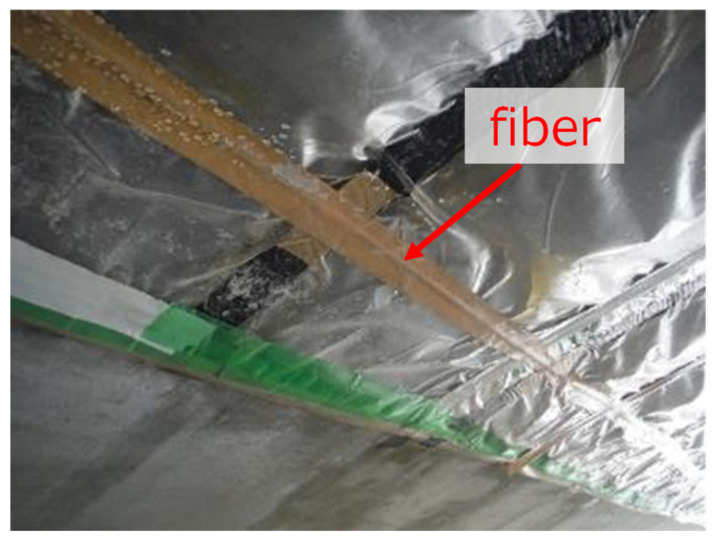
Fiber installation.

The sensing fiber was attached to the frame, and protected by the tape, as shown in Figure 25. The sides of the supporting frame were made from wood, which subjected to the weight of the injected concrete should deform more than in actual construction. This should allow poor filling to more likely occur at the shoulders’ proximity. The concrete was injected with a speed of 20 m^3^/h, and the maximum flow distance was 11.0 m.

**Figure 26:**
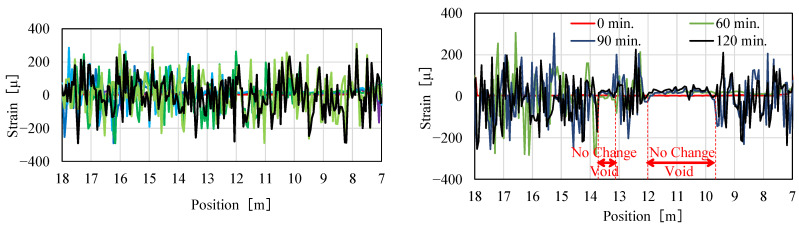
Selected strain change traces after 0, 60, 90, and 120 min from injection start.

During the entire period of concrete injection Rayleigh-based, strain change measurements were performed and the values were monitored in real-time. Figure 26 presents selected traces after 0, 60, 90, and 120 min for center (left) and shoulder (right) fibers.

**Figure 27:**
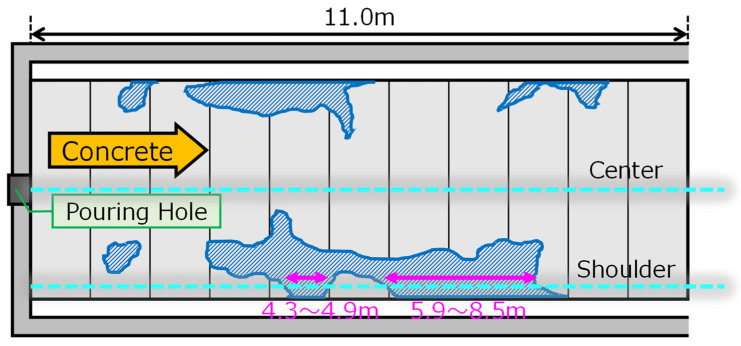
Location of voids based on inspection and that detected by optical fibers.

The front of the moving concrete was clearly detected on both fibers, so the progress of filling was observed. While the concrete completely filled the center part of the frame, there were two locations on the shoulder fiber with no response (strain change), indicating the location of voids. Those regions, from 4.3 to 4.9 m and from 5.8 to 8.5 m, are indicated in Figure 26 (right). Those locations were later confirmed by visual inspection and measurements as voids. The locations of all the voids and regions indicated by DFOS measurements are presented in Figure 27.

The full-scale mockup confirmed the reliability of strain change-based detection and its ability to provide real-time data to field engineers.

## 5. Conclusions

The large-scale installation of the optical fibers for sensing purposes is possible only during the construction phase of a project. At other phases of the project, it is either prohibitively expensive or technically impossible to make a proper optical fiber installation that is valuable for long-term Structural Health Monitoring. In the past, there was little interest from construction companies in using DFOS during the construction phases of a project. This has recently changed with the continued development and substantial improvements in DFOS technology on both sensing cables and interrogators. This has changed the landscape of SHM and the role of DFOS in that area of engineering. The key driver of change here is the Rayleigh Intensity Pattern technology, which has a precision better than 1 με over a long sensing distance range.

In this work, we have presented recent progress regarding real-time, on-site construction quality control monitoring using DFOS type-sensors. DFOS has the potential to replace point or point-wise sensors and continuously monitor values of strain and/or temperature along the entire structure. DFOS during the engineering stage of construction will change SHM, its perception, and most importantly, the attainable result in truly intelligent infrastructure design, engineering, and implementation.

## Figures and Tables

**Table 1:** Parameters of selected DFOS measurement methods.

Parameter	Feature	TW-COTDR	PSP-BOTDR	PPP-BOTDA	BOTDA	BOTDR	OBR (OFDR)
Strain accuracy, µε		0.1	6	10	20	50	1 *
Spatial resolution, cm	max	2	10	2	100	100	<1
	typical	10	20	10	100	200	1
Distance range, km	max	25	25	25	25	25	0.07
	typical	6	6	6	6	6	0.03

* Accuracy as provided by interrogator technical specifications.
